# Expression of Foxp3, CD25 and IL-2 in the B16F10 cancer cell line and melanoma is correlated with tumor growth in mice

**DOI:** 10.3892/ol.2013.1526

**Published:** 2013-08-13

**Authors:** D.F. MIRANDA-HERNÁNDEZ, M.A. FRANCO-MOLINA, E. MENDOZA-GAMBOA, P. ZAPATA-BENAVIDES, C.A. SIERRA-RIVERA, E.E. CORONADO-CERDA, A.G. ROSAS-TARACO, R.S. TAMÉZ-GUERRA, C. RODRÍGUEZ-PADILLA

**Affiliations:** 1 Department of Immunology and Virology, Faculty of Biological Sciences, University Autonoma of Nuevo León (UANL), San Nicolás de los Garza, Nuevo León 66450, Mexico; 2 Department of Immunology, Faculty of Medicine, University Autónoma of Nuevo León (UANL), Monterrey, Nuevo León 64460, Mexico

**Keywords:** forkhead box P3, interleukin-2, CD25, regulatory T cells, melanoma, cytokines

## Abstract

The forkhead box P3 (Foxp3) transcription factor is one of the most studied markers used to identify CD4^+^CD25^+^ regulatory T cells (Tregs), and has been identified as a key regulator in the development and function of Tregs. Foxp3 expression has been reported in a variety of solid human tumors, including melanoma. The aims of the present study were to analyze Foxp3 expression in B16F10 melanoma cells *in vitro*, to determine whether this expression was affected during tumor growth in a murine melanoma model and to correlate Foxp3 expression with CD25 expression, interleukin (IL)-2 production and tumor weight. Foxp3 expression was analyzed with quantitative (q)PCR, flow cytometry and confocal microscopy. CD25 expression was analyzed by flow cytometry, and cytokine production was measured by ELISA [IL-2, interferon (IFN)-γ, transforming growth factor (TGF)-β and IL-10] and flow cytometry (IL-2, IFN-γ, IL-4 and IL-5). Foxp3 and CD25 expression was detected in the B16F10 cells in culture and in the intratumoral B16F10 cells. An increase in Foxp3 and CD25 expression was observed in a time-dependent manner during tumor growth at 7, 14 and 21 days. The production of the IL-2, IL-10, IFN-γ and TGF-β cytokines was observed in the B16F10 cells and also detected in the tumoral microenvironment during tumor growth (7, 14 and 21 days). An increase in IL-2 and IL-10 production was observed, whereas IFN-γ production decreased in a time-dependent manner. The production of tumor necrosis factor (TNF)-α was not observed in culture, but was detected during tumor growth, whereas the production of IL-4 and IL-5 was not detected. These data showed a positive correlation between the expression of Foxp3, CD25 and IL-2 and tumor weight in murine melanoma. From these data, it may be suggested that Foxp3 participates in melanoma growth, the modulation of the IL-2, IFN-γ and TNF-α cytokines and CD25 expression, and that it also plays a possible role in immunosuppression.

## Introduction

Forkhead box P3 (Foxp3), a forkhead transcription factor family member implicated in T cell regulation, activation and differentiation, is a nuclear protein that is thought to function as a transcriptional repressor. The lack of a functional Foxp3 gene product has been demonstrated to lead to the defective production of regulatory T cells (Tregs), a subpopulation of T cells specialized in maintaining the balance between immunity and autotolerance (1). Increased Foxp3 expression in T cells (2), peripheral blood and tumors has been associated with disease progression and a worse prognosis in cancer patients, including melanoma patients. For a number of years, Foxp3 expression has been exclusively linked to the T cell lineage, but, recently, Foxp3 has been reported to be expressed in a variety of cancer cell lines (2–5). In a previous study, Foxp3 expression in primary breast cancer was associated with a worse overall survival probability, and this risk was correlated with increased Foxp3 immunostaining (6). Foxp3 expression in cancer cells may have a number of functions related to the evasion of the immune response, including the modulation of cytokines, chemokines, hormones and other proteins related to invasion and metastasis, but this remains to be examined. Understanding Foxp3 function is important for developing assays on the basis of using Foxp3 for disease prognosis, drug monitoring and treatment (2). In the present study, the levels of Foxp3, CD25 and interleukin (IL)-2 expression were investigated in the B16F10 cancer cell line *in vitro* and the expression levels of these factors were correlated with tumor growth in a murine model of melanoma.

## Materials and methods

### Animals and cell lines

The B16F10 murine melanoma cell line (American Type Culture Collection, Manassas, VA, USA) was cultured at 37°C in 5% CO_2_ in complete media consisting of Dulbecco’s modified Eagle’s medium (DMEM/F-12; Life Technologies, Invitrogen, Burlington, ON, Canada) and 10% fetal bovine serum (Gibco, Grand Island, NY, USA). C57/BL6 mice (5–6 weeks old) were acquired from Harlan Laboratories (Mexico). The mice were housed in a specific pathogen-free environment. All experimental protocols were approved by the Institutional Animal Care and Use Committee of the Department of Immunology and Virology of the University Autonoma of Nuevo León (UANL; Nuevo León, México).

### Foxp3 mRNA expression

Foxp3 mRNA expression was examined by quantitative (q)PCR. After total RNA isolation from the tumor cell line B16F10 and reverse transcription into cDNA, qPCR was performed using the Chromo4™ Real-Time PCR Detector (Bio-Rad, Hercules, CA, USA), the SYBR Supermix kit (Invitrogen, Paisley, UK), the RT^2^PCR Primer Set for Foxp3 (SuperArray Biosciences, Frederick, MD, USA) and β-actin as a reference gene (RT^2^PCR Primer Set; SuperArray Biosciences). The qPCR thermocycling conditions for Foxp3 were 10 min at 95°C for an initial hold, followed by 40 cycles of denaturation at 95°C, annealing at 60°C and extension at 72°C, all for 15 sec. The qPCR thermocycling conditions for β-actin were 10 min at 95°C for an initial hold, followed by 40 cycles of denaturation at 95°C for 15 sec and annealing/extension at 60°C for 60 sec. Relative expression was analyzed using Opticon Monitor™ software (Bio-Rad). For the PCR amplification, 20 μM of forward, 5′-GGCATCGTGATGGACTCCG-3′ and reverse, 5′-GCTGGAAGGTGGACAGCGA-3′ primers were used for β-actin, and the RT^2^PCR Primer Set was used for Foxp3 (SuperArray Biosciences) in a total reaction volume of 25 μl.

### Flow cytometry analysis

To analyze the cellular expression levels of CD25 and Foxp3, 1×10^6^ B16F10 cells obtained from culture or tumors were stained using an anti-mouse CD25 antibody (eBioscience, San Diego, CA, USA) in a final volume of 100 μl flow cytometry staining buffer and incubated at 4°C for 30 min. An Fc block was added to the incubation buffer with the anti-mouse CD25, and the cells were then washed in cold flow cytometry staining buffer. The cells were pelleted by centrifugation at 220 × g, then the supernatant was discarded and the cells were resuspended with a vortex pulse. Freshly prepared fixation/permeabilization working solution (1 ml) was added to the cells, which were then incubated at 4°C for 30 min, washed twice with 2 ml 1X permeabilization buffer and centrifuged at 440 × g to pellet the cells. The supernatant was then discarded, and Fc block in 1X permeabilization buffer was added to the cells in a final volume of 100 μl prior to incubation at 4°C for 15 min. An anti-mouse/rat Foxp3 (FJK-16s) antibody (eBioscience) in 1X permeabilization buffer was added without washing after the blocking step and incubated at 4°C for 30 min in the dark. Subsequently, the cells were washed once with 2 ml 1X permeabilization buffer. The stained cells were collected by centrifugation at 220 × g after discarding the supernatant and resuspended in 1% paraformaldehyde. The data were analyzed using a flow cytometer (Epics Altra; Beckman Coulter, Fullerton, CA, USA).

### Foxp3 detection by confocal microscopy

The B16F10 cells (1×10^6^) were treated with permeabilization solution (eBioscience) for 30 min at 4°C in the dark. Subsequently, the cells were centrifuged at 600 × g for 10 min and resuspended in 100 ml permeabilization buffer containing FITC-anti-mouse Foxp3 antibody (eBioscience) and incubated for 30 min. Subsequent to centrifugation, the cells were resuspended in 20 ml paraformaldehyde and mounted on the cover slide using Vectashield^®^ Mounting Medium with DAPI (Vector Laboratories, Burlingame, CA, USA) and immediately visualized by confocal microscopy.

### Tumor inoculation and B16F10 isolation

The B16F10 melanoma cells (5×10^5^) were resuspended in 200 μl phosphate-buffered saline (PBS) and subcutaneously inoculated into the right flank of 6-week-old C57BL/6 mice. The mice were sacrificed at 7, 14 and 21 days following a visible tumor appearance, and the tumor was removed. The B16F10 cells were obtained from the tumor by washing the tumor tissues with DMEM/F12 using a sterile syringe. Subsequent to being washed several times, 15 ml ammonia-chloride-potassium (ACK lysis buffer: 0.15 M NH_4_Cl; 0.1 mM KHCO_3_; 0.1 mM Na_2_EDTA, pH 7.2) was added to the collected cells to remove any red blood cells, and the cells were centrifuged at 440 × g. To eliminate the T cells and only obtain the intratumoral B16F10 cells, the cells were resuspended in isolation buffer from the Dynabeads^®^ FlowComp™ Mouse Pan T (CD90.2) kit (Invitrogen, Carlsbad, CA, USA) at a concentration of 1×10^8^ cells/ml. The FlowComp mouse CD90.2 antibody from the kit was added to the cell suspension at a ratio of 25 μl antibodies/500 μl cell suspension (5×10^7^ cells), then mixed and incubated for 10 min at 2–8°C. Following incubation, the cells were washed by adding 2 ml isolation buffer, then centrifuged at 440 × g for 8 min and resuspended in 1 ml isolation buffer. Resuspended Dynabeads FlowComp (75 μl) were added to the tube, mixed and incubated for 15 min at room temperature with rolling and tilting. The tube was then placed on a magnet for a minimum of 1 min. The supernatant was carefully collected while the tube remained on the magnet. The washing step was repeated once and the supernatant was collected again. Finally, the supernatant was cultured in a 25-cm^2^ cell culture flask and incubated in an atmosphere of 37°C and 5% CO_2_ for 3 h. Once the intratumoral B16F10 cells adhered to the flask, they were washed with PBS to remove any cell debris and unwanted cell types. The concentration of the intratumoral B16F10 melanoma cells was adjusted to 1×10^6^ cells in a final volume of 100 μl flow cytometry staining buffer (eBioscience). These cells were analyzed for Foxp3 and CD25 expression as described above.

### Isolation of intratumoral B16F10 melanoma cells

B16F10 melanoma cells (5×10^5^) resuspended in 200 μl PBS were subcutaneously inoculated into the right flank of 6-week-old C57BL/6 mice. Tumors were surgically removed at 7, 14 and 21 days after tumor appearance. The cells were collected after washing the tumor several times with DMEM/F12 using a sterile syringe. To lyse the red blood cells, ammonium-chloride-potassium (ACK) lysis buffer (0.15 M NH_4_Cl; 0.1 mM KHCO_3_; 0.1 mM Na_2_EDTA; pH 7.2) was added to the suspension of collected tumor cells, as previously described, in order to eliminate cell types that were not B16F10 cells. T lymphocytes from each pool of tumor cells were positively selected and removed using anti-CD90.2 mAb-coated Dynabeads, according to the standard immunoselection protocol recommended by the manufacturer (Dynal^®^, Invitrogen).

Finally, the cellular suspension was cultured in a 25-cm^2^ cell culture flask and incubated in an atmosphere of 37°C and 5% CO_2_ for 3 h. Once the intratumoral B16F10 cells had adhered to the flask, they were washed with PBS to remove any cell debris and other contaminating cells. The intratumoral B16F10 melanoma cells were adjusted to a concentration of 1×10^6^ cells in a final volume of 100 μl flow cytometry staining buffer (eBioscience). The cells were analyzed for Foxp3 expression as described previously.

### Cytokine production

The B16F10 melanoma cells (5×10^3^ cells/200 μl DMEM/F12 supplemented with 10% serum fetal bovine) were cultured for 48 h in an atmosphere of 37°C and 5% CO_2_, and the supernatants were collected and stored at −20°C for the subsequent analysis of cytokine production with ELISA [interferon (IFN)-γ, IL-2, transforming growth factor (TGF)-β and IL-10; Invitrogen]. The tumors induced by the B16F10 melanoma cells were collected by surgical means at 7, 14 and 21 days after tumor appearance and macerated by adding 1X PBS at a proportion of 400 mg tumor:1 ml PBS. The supernatants were collected and stored at −20°C until use in an ELISA for cytokine production (IFN-γ, IL-2, TGF-β and IL-10; Invitrogen) according to the manufacturer’s instructions. The absorbance was determined at 450 nm with a microplate autoreader (EL311; Bio Tek Instruments, Winooski, VT, USA). A commercial BD™ Cytometric Bead Array Mouse Th1/Th2 Cytokine kit (CBA; lot: 3817; BD Biosciences, San Diego, CA, USA) was used to determine the levels of IL-2, IFN-γ, IL-4, IL-5 and tumor necrosis factor (TNF)-α (pg/ml) in supernatants of the B16F10 melanoma and tumor cells, according to the manufacturer’s instructions. Fluorescence was analyzed using a flow cytometer (BD Accuri C6; Becton-Dickinson Biosciences, Ann Arbor, MI, USA), and the cytokine level was determined using FCAP Array Software V 3.0 (Soft Flow Hungary, Ltd., Pécs, Hungary). The parameters were determined in the supernatants obtained after 48 h of B16F10 cell culture and in the tumors of the mice with melanoma, which were collected at 7, 14 and 21 days after the appearance of the tumor.

## Results

### Foxp3 detection in B16F10 cells

**Figure 1:**
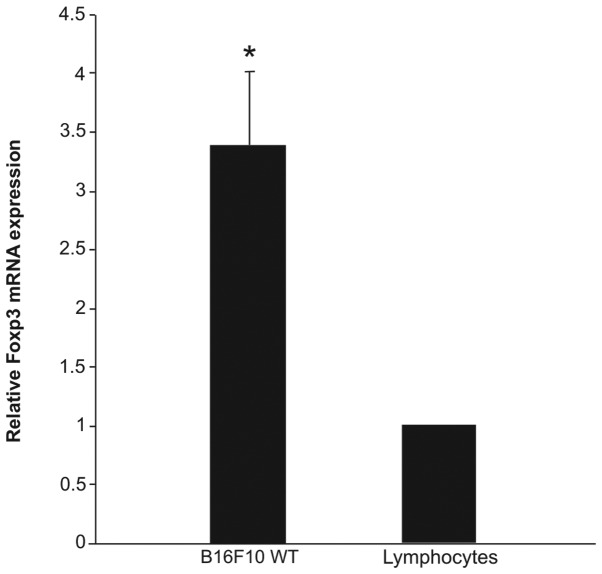
Expression of Foxp3 mRNA. Foxp3 mRNA in B16F10 melanoma cancer cells was analyzed by quantitative (q)PCR and normalized to β-actin as an endogenous control and murine lymphocytes as a positive control. The data represent the mean number from three repeat samples (^*^P<0.005 vs. lymphocyte control). Foxp3, forkhead box P3.

**Figure 2:**
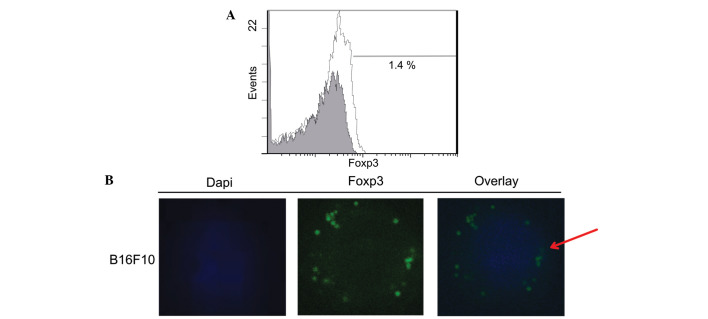
Foxp3 expression in B16F10 murine melanoma cells. (A) B16F10 cells were cultured for 48 h, then collected, permeabilized, stained for Foxp3 and analyzed by flow cytometry. The percentage of Foxp3^+^ cells is shown. The results are representative of four separate experiments. The white curve represents specific staining for Foxp3, and the gray curve represents the isotype control. (B) B16F10 melanoma cancer cells were fixed and permeabilized prior to Foxp3 staining (green). The nuclei were counterstained with DAPI (blue) and analyzed by confocal microscopy. The red arrows indicates Foxp3.

The expression of Foxp3 was detected in the B16F10 melanoma cells at the mRNA (Fig. 1) and protein (Fig. 2) levels. The results of qPCR demonstrated that the Foxp3 expression was 2.5-fold higher in B16F10 cells than in the murine lymphocytes used as a positive control (Fig. 1) and flow cytometry revealed that the protein expression level was 1.4% (Fig. 2A). These results were confirmed with double-label immunofluorescence confocal microscopy analysis (Fig. 2B).

### Cytokine production

The production of the following cytokines was detected in the cultured B16F10 cells by ELISA: IL-10 (1.75 pg/ml), IL-2 (86.41 pg/ml), IFN-γ (2.45 pg/ml) and TGF-β (2.85 pg/ml).

The cytokine production from tumors at 7, 14 and 21 days demonstrated increasing amounts of IL-10 (0.53, 1.36 and 3.90 pg/ml, respectively) and IL-2 (4.06, 9.93 and 18.89 pg/ml, respectively), whereas IFN-γ production decreased (20.38, 13.65 and 4.45 pg/ml, respectively) and the production of TGF-β was not affected (1.27, 1.18 and 1.89 pg/ml, respectively; P<0.05; Table I). When cytokine production was examined by flow cytometry, the IL-2 levels in the B16F10 cell line and at 7, 14 and 21 days of tumor growth were 45.72 pg/ml and 4.88, 7.37 and 13.55 pg/ml, respectively, and the IFN-γ levels were 1.05 pg/ml and 7.02, 3.75 and 0.95 pg/ml, respectively. TNF-α was not detected in the B16F10 cell line, but was detected in the tumor at 1.92, 2.32 and 3.77 pg/ml, respectively. IL-4 and IL-5 were not detected in the cell line or the tumor (P<0.05; Table I).

### Foxp3 and CD25 expression in intratumoral B16F10 cells

**Figure 3:**
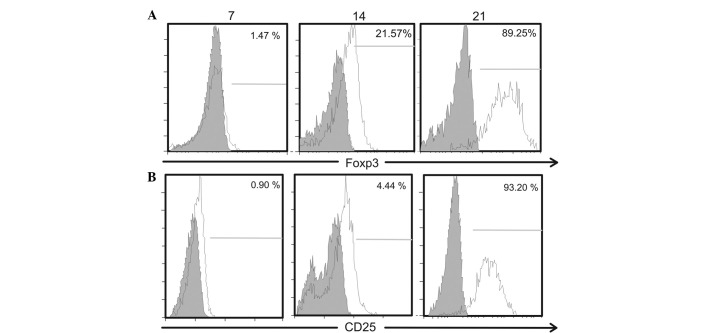
Analysis of Foxp3 and CD25 expression in intratumoral B16F10 cells during the development of murine melanoma. Tumors were collected at 7, 14 and 21 days after tumor appearance, and intratumoral B16F10 murine melanoma cells were obtained as described in the Materials and methods. (A) The intratumoral B16F10 melanoma cells were adjusted and stained with PE-Cy5 for the Foxp3 analysis. (B) For the CD25 analysis, intratumoral B16F10 cells were stained with PE and analyzed by flow cytometry. The white curve represents specific staining for CD25^+^ cells, and the gray curve represents the isotype control. The numbers in the histograms indicate the percentage of CD25^+^ cells. Foxp3, forkhead box P3; PE, phycoerythrin.

**Figure 4:**
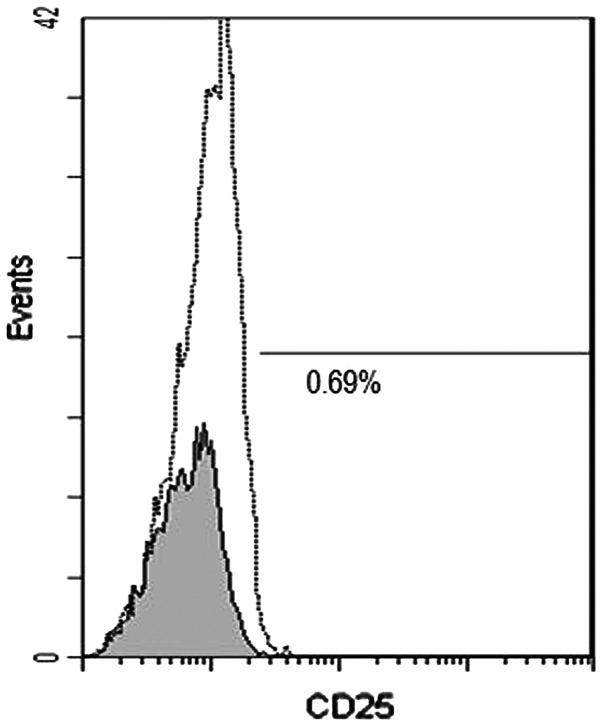
CD25 expression in B16F10 murine melanoma cells. (A) B16F10 cells were cultured for 48 h, then collected, stained for CD25 and analyzed by flow cytometry. The percentage of CD25^+^ cells is shown, and the result is representative of three separate experiments. The white curve represents specific staining for CD25^+^ cells, and the gray curve represents the isotype control.

An increase was observed in Foxp3 expression during tumor growth in a time-dependent manner by flow cytometry at 7 (1.47%), 14 (21.57%) and 21 (89.25%) days (Fig. 3A and Table II). Due to the high production of IL-2 in the B16F10 cells *in vitro*, the expression of CD25 was evaluated. It was demonstrated that 0.69% of the cellular population expressed this marker *in vitro* (Fig. 4), while *in vivo*, CD25 expression was detected at 7 (0.90%), 14 (4.44%) and 21 (93.20%) days of tumor growth in the B16F10 cells (Fig. 3B and Table II).

### Correlation between days of tumor growth, Foxp3, IL-2, CD25 and tumor weight

**Figure 5:**
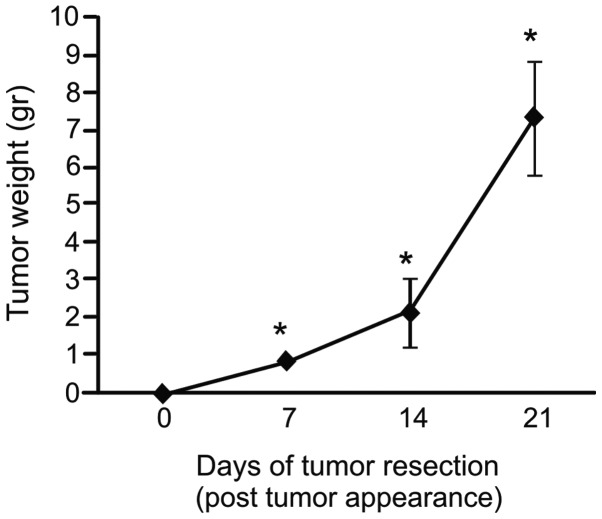
Tumor weight in a murine melanoma model. B16F10 cells (5×10^5^ cells/200 μl) were subcutaneously injected into the right flank of C57BL6 mice. The tumors were visible at day 7 after the cells were injected and collected at days 7, 14 and 21 after tumor appearance. The data represent the mean of 3 murine melanoma tumor weights for each day of tumor resection. ^*^P<0.05 vs. control.

A significant correlation was observed between the tumor weight (Fig. 5), Foxp3 expression, IL-2 production and CD25 expression, which was dependent on the days of tumor growth, with the exception of the correlation of IL-2 production with CD25 expression at 14 days (r^2^=0.832) and IL-2 production with tumor weight at 21 days (r^2^=0.688) (Table III).

## Discussion

Melanoma is a highly aggressive form of cancer. A tumor thickness approaching 4 mm presents a high risk of metastasis, and patients diagnosed with metastatic melanoma have a median survival of 6–9 months (7). Surgery eradicates so-called thin melanomas, but in a significant number of patients with cutaneous melanomas that are 2–4 mm-thick, the melanomas recur at local or distant sites post-surgery. Therefore, the development of strategies aimed at identifying biochemical markers, which indicate potentially early lesions that may develop into highly metastatic tumors, and the appropriate targets for drug intervention, is of crucial clinical importance. Previous studies indicated that Foxp3 is expressed in breast cancer cells, and that the expression level was associated with patient survival (6). The expression of Foxp3 in tumor cells has also been recently reported in pancreatic cancer, melanoma and other tumor cell lines. Hinz *et al*(8) reported that Foxp3 is expressed in pancreatic cancer cells, and that Foxp3-expressing cancer cells inhibited the proliferation of CD4^+^CD25^−^ T cells, potentially contributing to the immune evasion of the tumor cells. Ebert *et al*(9) reported the expression of Foxp3 in not only Tregs, but also in melanoma cells in metastatic melanoma tissue and tumor cell lines derived from melanoma (SK-Mel-1 and SK-Mel-28) and other solid tumors [A172 and U-87 MG (glioma); DU 145, PC-3 and LN-CaP (prostate cancer); A549, CaLu-6 and NCI-H460 (lung cancer); HCT116, Caco-2 and SW480 (colorectal cancer); HT-1376, HT-1197 and HT-5637 (bladder cancer); and MCF7, MDA-MB-231 and MDA-MD-468 (breast cancer)] (10). The present study showed that the FoxP3 transcription factor is expressed by the B16F10 cell line and B16F10 intratumoral cells. This observation is particularly notable, as the present data corroborate the findings by Ebert *et al*(9) in melanoma cutaneous tissues, cell lines and the studies mentioned above. These results from B16F10 melanoma cells suggest a major role of Foxp3 in melanoma growth and may support the design of experimental strategies using RNA interference to inhibit tumor growth and enhance immunogenicity or, alternatively, to vaccinate against the Foxp3 Δ3,4 isoform. This hypothesis should be examined in experiments in which Foxp3 expression is knocked down in a B16F10 cell line. These cells may subsequently be used to inoculate a murine model to corroborate this approach. The B16F10 cell line was also demonstrated to produce IL-2, IL-10, IFN-γ and TGF-β cytokines in the present study, and this cytokine production was also detected in the tumoral microenvironment in a cytokine-dependent (IL-2 increase and IFN-γ decrease) and time-dependent manner induced by inoculation of these cells in mice. A previous study observed the production of cytokines in cancer cell lines (PANC-89 expresses IL-6 but not TGF-β, whereas PANC-1 expresses TGF-β but not IL-6) (10). The present study corroborated the production of IL-2 and IFN-γ by flow cytometry in the B16F10 cells in culture and also analyzed CD25 expression. This receptor was expressed in B16F10 cells and B16F10 cells derived from the tumor, and its expression was increased in a manner dependent on tumor growth. The role of Foxp3 in human tumor cells may vary among different tumor cell types. This variation may be as Foxp3, as a suppressive transcription factor, represses different molecular targets in various cells, and the fact that Foxp3 may regulate the expression of several cytokines and their receptors, including IL-2 and CD25 in B16F10 cells. These factors play important roles in the immunosuppression of cancer as the decreased production of cytokines that play roles in enhancing tumor immunity and the increased production of cytokines that participate in immunosuppression, including IL-10 and IL-2, play a role in the generation of Treg cells. However, this hypothesis requires experimental studies in which antibodies against IL-2 and CD25 and cyclosporine are added to B16F10 cells to determine the level of cellular proliferation. From the present data, it may be suggested that Foxp3 participates in tumor growth and the modulation of the IL-2, IFN-γ and TNF-α cytokines and CD25, and that it may play a role in the immunosuppression of melanomas, possibly via this pathway.

## Figures and Tables

**Table I:** Cytokine production in B16F10 murine melanoma cells *in vitro* and from tumor supernatants of murine melanoma.

Assay	Cytokines	Cytokine production			

		B16F10 cell supernantants *in vitro* 48 h	B16F10 tumor supernatants		

			7 days	14 days	21 days
ELISA	IL-10	1.750±0.002	0.530±0.007	1.360±0.0073	3.901±0.435
	IL-2	86.410±5.080^a^	4.060±0.233	9.933±2.053^a^	18.893±2.438^a^
	TGF-β	2.850±0.305	1.270±0.410	1.180±0.862	1.895±0.196
	INF-γ	2.450±0.001	20.384±1.200	13.653±1.796^a^	4.450±1.990^a^
Flow cytometry	IL-2	45.720±1.740	4.883±0.174	7.370±1.241	13.550±1.148^a^
	INF-γ	1.050±0.212	7.020±0.530	3.754±0.742^a^	0.953±0.092^a^
	TNF-α	ND	1.922±0.084	2.322±0.183	3.778±0.160^a^
	IL-4	ND	ND	ND	ND
	IL-5	ND	ND	ND	ND

Cytokine production in B16F10 murine melanoma cells *in vitro* and from tumor supernatants of murine melanomas collected at 7, 14 and 21 days after tumor appearance, as measured by ELISA and flow cytometry assays. The total protein was adjusted to 1 mg prior to analysis. The data are expressed as the means of three independent experiments in B16F10 melanoma cells (n=3) and the mean cytokine production in supernatants of murine melanoma tumors collected during melanoma growth *in vivo*. Data are presented as mean ± standard deviation.

a P<0.05 indicates a significant difference in cytokine production by ELISA and flow cytometry in supernatants *in vitro* and a significant difference between the days of melanoma development.

ND, no detected production; IL, interleukin; IFN-α, interferon-α; TGF-α, transforming growth factor-α; TNF-α, tumor necrosis factor-α.

**Table II:** Determination of IL-2 production, CD25 and Foxp3 expression in intratumoral B16F10 cells during tumor growth in a murine melanoma model *in vivo*.

Tumor development, days	IL-2 production, pg/ml	CD25 expression, %	Foxp3 expression, %
7	4.06±0.24	0.90	1.47
14	9.93±2.05^a^	4.44^a^	21.51^a^
21	18.89±2.43^a^	93.20^a^	89.25^a^

IL-2 production values presented as mean ± standard deviation.

a P<0.05.

IL, interleukin; Foxp3, forkhead box P3.

**Table III:** Correlation coefficients of intratumoral B16F10 Foxp3 expression, IL-2 production, B16F10 CD25 surface expression and tumor weight (g) during murine melanoma development.

	Correlation coefficient at 14 days				Correlation coefficient at 21 days			
		
7 days	Intratumoral Foxp3	IL-2	CD25	Tumor weight, g	Intratumoral Foxp3	IL-2	CD25	Tumor weight, g
Intratumoral Foxp3	0.998^a^	0.887	0.997^a^	0.998^a^	0.999^a^	0.870	0.998^a^	0.995
IL-2	0.893	1.000^b^	0.832	0.949	0.893	1.000^b^	0.982^a^	0.688^a^
CD25	0.998^a^	0.923^a^	0.982	0.997^a^	0.995^a^	0.951^a^	0.999^a^	0.915
Tumor weight	0.999^a^	0.873	0.993^a^	0.982^a^	0.961^a^	0.870	0.998^a^	0.955^a^

a P<0.05 and

b P<0.01.

IL, interleukin; Foxp3, forkhead box P3.

## Abbreviations

Tregs: regulatory T cells
IL: interleukin
IFN: interferon
TGF: transforming growth factor
TNF: tumor necrosis factor
